# Interactions between Polygenic Risk Scores, Dietary Pattern, and Menarche Age with the Obesity Risk in a Large Hospital-Based Cohort

**DOI:** 10.3390/nu13113772

**Published:** 2021-10-25

**Authors:** Sunmin Park, Hye Jeong Yang, Min Jung Kim, Haeng Jeon Hur, Soon-Hee Kim, Myung-Sunny Kim

**Affiliations:** 1 Obesity/Diabetes Research Center, Department of Food and Nutrition, Hoseo University, Asan-si 31499, Korea; 2 Research Group of Healthcare, Korea Food Research Institute, Wanju-gun 55365, Korea; yhj@kfri.re.kr (H.J.Y.); kmj@kfri.re.kr (M.J.K.); mistltoe@kfri.re.kr (H.J.H.); shkim@kfri.re.kr (S.-H.K.); 3 Department of Food Biotechnology, Korea University of Science & Technology, Wanju-gun 55365, Korea

**Keywords:** nutrigenomics, skeletal muscle index, obesity, menarche age, plant-based diet, fried foods

## Abstract

Obese Asians are more susceptible to metabolic diseases than obese Caucasians of the same body mass index (BMI). We hypothesized that the genetic variants associated with obesity risk interact with the lifestyles of middle-aged and elderly adults, possibly allowing the development of personalized interventions based on genotype. We aimed to examine this hypothesis in a large city hospital-based cohort in Korea. The participants with cancers, thyroid diseases, chronic kidney disease, or brain-related diseases were excluded. The participants were divided into case and control according to their BMI: ≥25 kg/m^2^ (case; *n* = 17,545) and <25 kg/m^2^ (control; *n* = 36,283). The genetic variants that affected obesity risk were selected using a genome-wide association study, and the genetic variants that interacted with each other were identified by generalized multifactor dimensionality reduction analysis. The selected genetic variants were confirmed in the Ansan/Ansung cohort, and polygenetic risk scores (PRS)−nutrient interactions for obesity risk were determined. A high BMI was associated with a high-fat mass (odds ratio (OR) = 20.71) and a high skeletal muscle-mass index (OR = 3.38). A high BMI was positively related to metabolic syndrome and its components, including lipid profiles, whereas the initial menstruation age was inversely associated with a high BMI (OR = 0.78). The best model with 5-SNPs included *SEC16B*_rs543874, *DNAJC27*_rs713586, *BDNF*_rs6265, *MC4R*_rs6567160, and *GIPR*_rs1444988703. The high PRS with the 5-SNP model was positively associated with an obesity risk of 1.629 (1.475–1.798) after adjusting for the covariates. The 5-SNP model interacted with the initial menstruation age, fried foods, and plant-based diet for BMI risk. The participants with a high PRS also had a higher obesity risk when combined with early menarche, low plant-based diet, and a high fried-food intake than in participants with late menarche, high plant-based diet, and low fried-food intake. In conclusion, people with a high PRS and earlier menarche age are recommended to consume fewer fried foods and a more plant-based diet to decrease obesity risk. This result can be applied to personalized nutrition for preventing obesity.

## 1. Introduction

Asians with a high body mass index (BMI) have a higher body fat mass than Caucasians. Asians with higher adiposity have a greater risk of metabolic diseases than Caucasians with the same adiposity level [1]. Accordingly, the World Health Organization defines obesity among Asians as a BMI of 25 kg/m^2^ or more for both genders and as a body fat of 25% and 30% for men and women, respectively [2].

Obesity is a multifactorial disorder including genetic, gut microbial, and environmental factors that influence energy balance [3]. Although monogenic variants are involved in severe obesity, they are rare, and approximately 500 genetic variants of approximately 400 genes have been revealed to be associated with obesity risk at *p* < 5 × 10^−8^ [4]. Most studies have explored the effect of an individual genetic variant on obesity risk [4,5,6,7]. A polygenic model provides the cumulative genetic impact on obesity risk compared to individual genetic variants when the selected genes have gene−gene and SNP−SNP interactions with the pathways of biological relevance to obesity [8]. Polygenetic risk scores (PRS) calculated by a sum of the number of risk alleles of each SNP is informative for examining the polygenetic impact on obesity risk [5,6]. Fat mass and obesity-associated protein (*FTO*), melanocortin 4 receptor (*MC4R)*, and brain-derived neurotrophic factor (*BDNF)* genetic variants have been reported to be strongly associated with obesity risk in a genome-wide association study (GWAS) and meta-analysis studies of children and adults of different ethnicities including Europeans, Americans, Asians, and Africans [8,9,10,11,12]. 

In the protein−protein interaction network analysis, *MC4R*, one of the hub genes related to obesity, is highly interconnected with gastric inhibitory polypeptide receptor (*GIPR),* luteinizing hormone/choriogonadotropin receptor *(LHCGR),* calcitonin receptor *(CALCR),* adenylate cyclase (*ADCY)3, ADCY9, FTO,* transmembrane protein 18 (*TMEM18), BDNF,* and potassium channel tetramerization domain containing 15 (*KCTD15)* [4]. These genes are related to the obesity-related signaling pathways such as the AMP kinase (AMPK), neurotrophin, and phosphoinositide 3-kinases àAkt signaling pathway. Their variants affect the obesity risk by modulating the binding affinity of obesity-related transcription factors, such as *STAT3*, *CEBPB*, *TCF7L2*, *FTO*, and *GATA2*, and changing the phosphorylation of proteins, such as BDNF with the rs6265 risk allele. The interaction of the genetic variants may account for a more significant proportion of the genetic impact on obesity. However, the genetic variant−genetic variant interaction of the obesity-related genes has not been highly studied.

Furthermore, obesity-related genetic variants do not always equally influence obesity risk in different people because there are complex interactions between genetic and environmental factors, behavior, and the gut microbiota, influencing the actual obesity phenotypes. Recent studies have demonstrated that an individual genetic variant and lifestyle interactions influence the risk of obesity [13] and metabolic diseases [14,15] in Europeans and Asians [16]. For example, *FTO* genetic variants interact with the genetic and environmental factors that influence obesity risk [17,18]. Adult Asians with the *FTO*_rs1421085 risk allele have a higher BMI than those with the nonrisk allele when they have no regular exercise. On the other hand, adults with the risk allele have a lower BMI when they engage in regular exercise [19]. These observations suggest that the interaction of regular exercise with *FTO*_rs1421085 modulates obesity risk, indicating that genetic variants interact with lifestyles. However, there is no study to determine the interaction between the PRS of the genetic variant−genetic variant interaction and lifestyles. This study hypothesized that the PRS of the interacting genetic variants associated with obesity risk interacts with lifestyle factors in middle-aged and elderly adults. We aimed to examine the hypothesis in a large city hospital-based cohort in Korea.

## 2. Methods

### 2.1. Participants

During 2004–2013, 58,630 Korean adults aged >40 years volunteered to participate in a hospital-based city cohort study called the Korean Genome and Epidemiology Study (KoGES) organized by the Korean Centers for Disease Control and Prevention. The replicate study, used only to validate obesity-related genetic variants, was conducted in 5493 adults, aged 40–79 years, for whom Korean Chip data was available among the Ansan/Ansung cohort. People with a history of cancers, thyroid diseases, chronic kidney disease, and brain-related diseases were excluded from the participants. The urban hospital-based cohort and Ansan/Ansung cohort excluded 4802 and 691 patients, respectively. All procedures of the KoGES conformed with the Declaration of Helsinki and were approved by the Institutional Review Boards of the Korean National Institute of Health (KBP-2019-055) and Hoseo University (1041231-150811-HR-034-01). All participants provided written informed consent.

### 2.2. Demographic, Anthropometric, and Biochemical Information

Participants were interviewed to obtain their demographic information (e.g., age, residence area, education, income, current occupation, smoking status, alcohol intake, and regular exercise) [20]. The residence area was where the participant had lived for over 6 months when he/she enrolled in the city-based cohort study. It was categorized into six provinces, including Gyeonggi-do plus Seoul, Chungcheong-do, Gangwon-do, Jeolla-do, Gyeongsang-do plus Busan, and Jeju-Do. Household income was stratified as very low (<USD 1000/month), low (USD 1000–2000/month), intermediate (USD 2000–4000/month), or high (>USD 4000/month) [21]. Educational status was classified as less than high school, high school, or college or higher. Current job status was defined as the occupation where the person had been employed for the longest period if the job was recently changed. Current job status was classified as unemployed when a participant reported being unemployed or a housewife and employed as professional, director, office worker, service staff, salesperson, agricultural or fishery worker, labor worker. Alcohol intake was quantified from the questionnaires about alcohol drinking frequencies and amounts according to alcohol beverage types. The participants were classified as light (<100 g/week) or heavy (≥100 g/week) drinkers based on their average daily alcohol intake (Table 1) [21]. The smoking status was categorized as current, past, or never, and defined as current when >100 cigarettes had been smoked over the previous six months [21].

Anthropometric measurements, including body weight, height, and waist circumferences, were assessed according to standardized procedures [22]. After an overnight fast (no food for over 12 h), a participant visited the hospital, and they switched clothes into a light gown with no shoes. Body weight, and height were measured in a standing position using a well-calibrated digital weight and height scale (Inbody, Cheonan, Korea). Waist circumference was measured taken around the abdomen at the position of two finger-widths above the umbilicus in a relaxed state with a tape measure (Stanley, New Britain, CT, USA). The BMI was calculated by dividing weight (kilograms) by height^2^ (meters). Blood pressure was measured using a sphygmomanometer (W.A. Baum Co., New York, NY, USA), on the right arm, in a sitting position, at heart level. The biochemical parameters were determined using plasma and serum from blood drawn after an overnight fast [22]. Skeletal muscle mass was predicted using a machine learning approach from the Ansan/Ansung cohort data measured with an Inbody scanner (Cheonan, Korea) [23]. The skeletal muscle index was defined as skeletal muscle mass divided by the BMI [23]. The lipid profiles and the glucose and creatinine in plasma and serum concentrations were measured using a Hitachi 7600 Automatic Analyzer (Hitachi LTD., Tokyo, Japan). WBC counts were obtained from heparin-treated blood. The blood hemoglobin A1c (HbA1c; glycated hemoglobin) concentrations were determined using a Hitachi 7600 Automatic Analyzer (Hitachi, Tokyo, Japan), and the plasma lipid profiles (total cholesterol, HDL, and triglyceride) were examined using a Hitachi 7600 Automatic Analyzer. The serum high-sensitive C-reactive protein (hs-CRP) concentrations were measured using an ELISA kit. The estimated glomerular filtration rate (eGFR) was calculated using the equation of 175 × (serum creatinine concentrations)^−1.154^ × (age)^−0.203^. In females, the eGFR was multiplied by 0.742.

### 2.3. Definition of Obesity and MetS

Obesity for Asians is defined as ≥25 kg/m^2^ [24]. In the urban hospital-based and Ansan/Ansung cohorts, 17,545 and 2756 participants belonged to high-BMI (case), respectively, and 36,283 and 5395 participants in low-BMI (control), respectively.

MetS was defined according to the 2005 revised National Cholesterol Education Program−Adult Treatment Panel III criteria for Asia [16,17]. Participants with three or more of the following criteria were considered as having MetS: (1) elevated blood pressure (average systolic blood pressure ≥130 mmHg or diastolic blood pressure ≥85 mmHg) or current blood pressure medication use; (2) low HDL-C concentration (<40 mg/dL for men and <50 mg/dL for women); (3) elevated serum triglyceride concentration (≥150 mmol/L) or current anti-dyslipidemic medication use; (4) elevated fasting blood glucose concentration (≥100 mmol/L) or current use of antidiabetic medication; (5) abdominal obesity (waist circumference ≥ 90 cm for men and ≥85 cm for women).

### 2.4. Food and Nutrient Intake and Dietary Patterns

The food and nutrient intakes were assessed using a semi-quantitative food frequency questionnaire (SQFFQ) developed and validated during the KoGES [25]. The usual dietary intake was estimated over the previous six months. The questionnaire requested information regarding the intake of 106 food items. Each participant completed the SQFFQ, and 23 nutrient intakes were calculated from the SQFFQ data using the Computer-Aided Nutritional Analysis Program (CAN Pro) 3.0, a nutrient database program developed by the Korean Nutrition Society [25].

The 106 food items included were categorized into 29 food groups. These 29 food groups were used as independent variables during factor analysis to determine the dietary patterns using the FACTOR procedure. The number of factors retained in principle component analysis was determined using the eigenvalues >1.5, and the orthogonal rotation procedure (Varimax) was applied [15]. Dietary factor-loading values of ≥0.40 indicated significant contributions to the dietary patterns. Four distinct dietary factors were selected for the Korean dietary patterns. As integrating food intake estimated by SQFFQ, the participants were categorized into a balanced Korean diet, plant-based diet, Western-style diet, and rice-based diet. The plant-based diet was high in beans, eggs, milk, beverage, fruits, and nuts, while the Western-style diet was rich in noodles, soups, meats, processed meats, and fast foods.

### 2.5. Dietary Inflammatory Index (DII)

DII represents an index of dietary inflammatory potentials from individual food and nutrient intake using their dietary inflammatory weights for certain foods and nutrients (energy, 32 nutrients, four food products, four spices, and caffeine), as previously described [26]. Since the SQFFQ did not include the intake of spices, we excluded intake of garlic, ginger, saffron, and turmeric from DII calculations. DII was calculated by multiplying the dietary inflammatory scores of the 38 food and nutrient components by daily intake and dividing the sum of the scores of 38 items by 100.

### 2.6. Genotyping Using a Korean Chip and Quality Control

The genotype data were provided by the Center for Genome Science at the Korea National Institute of Health. Genomic DNA was isolated from whole blood, and genotypes were determined using a Korean Chip (Affymetrix, Santa Clara, CA, USA), which was designed to examine the Korean genetic variants and included the disease-related single nucleotide polymorphisms (SNPs) [27]. The genotyping accuracy was checked by Bayesian Robust Linear Modeling using the Mahalanobis Distance Genotyping Algorithm [28]. For genotyping analysis using this Korean chip, the genotyping accuracy, missing genotype call rate, and heterozygosity were ≥98%, <4%, and <30%, respectively; the data showed no gender bias. In addition, genetic variants that met the Hardy−Weinberg equilibrium (HWE, *p* > 0.05) and minor allele frequency (MAF) > 1% criterion were included [28].

### 2.7. Genetic Variants Influencing the Obesity Risk and the Best Model with SNP−SNP Interactions

**Figure 1:**
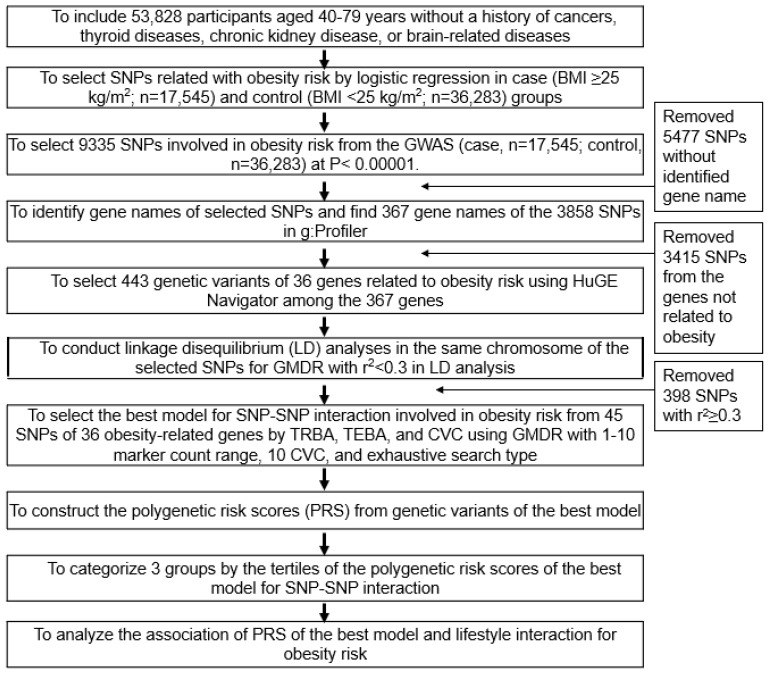
Flow chart for the generation of polygenetic variants that increase the risk of obesity and interactions between polygenetic risk scores (PRS) and lifestyles.

The steps to select genetic variants for the best model of SNP−SNP interactions are shown in Figure 1. Genetic variants for obesity risk were explored by conducting GWAS with low-BMI (*n* = 36,283) and high-BMI groups (*n* = 17,545) in the urban hospital-based cohort (*p* < 0.00001). From the GWAS, 9335 genetic variants were selected at *p* < 5 × 10^−5^. Gene names of the 9335 SNPs were identified using g:Profiler (https://biit.cs.ut.ee/gprofiler/snpense, accessed on 29 March 2021), and 367 genes (3858 SNPs) were identified. The 3415 SNPs without identified gene names were discarded since their association with obesity cannot be explained by associations with obesity-related pathways. Among the 367 genes, the 36 obesity-related genes (453 SNPs) based on the literature were selected using HuGE Navigator (https://phgkb.cdc.gov/PHGKB/hNHome.action, accessed on 8 April 2021). The corresponding linkage disequilibrium (LD) analyses were performed on the SNPs of the selected genes in the same chromosomes using Haploview 4.2 in PLINK. Because SNPs with high r^2^ values provided the same information on the genetic impact, they were not included in the GMDR. The potential genetic variants in the same chromosome were not strongly correlated in an LD analysis (r^2^ < 0.3).

Of the 45 potential genetic variants in the 36 obesity-related genes, ten SNPs exhibiting SNP−SNP interactions strongly associated with an obesity risk were automatically selected by generalized multifactor dimensionality reduction (GMDR) with 1–10 marker count range, 10 CVC, and exhaustive search type. GMDR provided ten potential models among different combinations of 45 genetic variants. The best SNP−SNP interaction model was selected in a sign rank test of trained balanced accuracy (TRBA) and testing balanced accuracy (TEBA) with or without adjusting for the covariates using a GMDR program and a *p*-value threshold of 0.05 [29]. The covariates used were age, gender, residence area, education, income, occupation, energy intake, alcohol intake, regular exercise, and smoking status. Ten-fold cross-validation was also checked for cross-validation consistency (CVC) because the sample size was larger than 1000 [29], and 10 out of 10 in CVC met the perfect cross-validation criteria. Using the best model determined by GMDR analysis, the risk allele of each SNP in the selected best model was counted as 1. For example, when people had AA, AG, and GG of one SNP, and the A allele was the risk allele, the genetic score for the SNP was 2, 1, and 0, respectively. The PRS for the best gene−gene-interaction model was assessed by summating the number of the risk alleles (genetic score) from each selected SNP in the best gene−gene-interaction model [5,6,30]. The PRS in the five and seven SNP models was divided into three categories according to the number of risk alleles; they were classified as low PRS, middle PRS, and high PRS when the number of risk alleles in the PRS was 0–3, 4–5, and ≥6 in the 5-SNP model and 0–5, 6–7, and ≥8 in the 7-SNP model, respectively.

### 2.8. Statistical Analyses

Statistical analysis was conducted using PLINK version 2.0 (http://pngu.mgh.harvard.edu/~purcell/plink, accessed on 9 March 2021) and SAS version 9.3 (SAS Institute, Cary, NC, USA). Descriptive statistics of the categorical variables (e.g., gender and smoking status, etc.) were analyzed using the frequency distributions in the low-, middle-, and high-PRS groups. The significant differences between their frequency distributions were assessed using a chi-squared test. The descriptive values of continuous variables were expressed as the means and standard deviations according to the PRS categories. The significance of the differences among the PRS groups was analyzed using a one-way analysis of variance (ANOVA) to adjust covariates. Finally, multiple comparisons among the PRS groups were performed using a Tukey’s test.

The associations among the PRSs were obtained using the best model, and the obesity risk was examined using multivariate logistic regression analysis with an adjustment for covariates. The odds ratios (ORs) and 95% confidence intervals (CI) were calculated against an index reference: low PRS. Multivariate logistic regression analysis was performed using two adjusted models. The covariates of model 1 included gender, age, residence area, education, and income, while those of model 2 contained the covariates in model 1 plus smoking status, drinking amount, daily energy intake, and regular exercise. The adjusted ORs and 95% CI were calculated for obesity risk according to PRS.

The participants were categorized into higher and lower intake groups using the classification criterion described above. A multivariate interaction model was used to examine the interactions between PRS and lifestyles and demographic parameters after adjusting for the covariates. *p* values < 0.05 were considered significant. 

## 3. Results

### 3.1. General and Demographic Characteristics of the Participants According to Gender and Obesity

The men were older than the women, while the obese women were older than the nonobese women (*p* < 0.001). Obese women were shorter than nonobese women, but there was no height difference linked to obesity in men (*p* < 0.001). The average BMIs of the nonobese and obese groups were approximately 22.5 and 27.1 kg/m^2^, respectively, in both gender groups (Table 1). The waist circumferences were higher in the obese group than the nonobese group in both genders, but men in the obese and nonobese groups had greater waist circumferences than women in the corresponding weight groups. Interestingly, the skeletal muscle and fat mass were higher in the obese group than the nonobese group for both genders (*p* < 0.05; Table 1). On the other hand, the adjusted ORs were much higher for waist circumferences (OR = 18.3 and 95% CI: 16.5–20.3) and fat mass (OR = 20.71 and 95% CI = 17.83–24.16) for obesity risk than the skeletal muscle mass. The initial menstrual age was younger in the obese group than in the nonobese group, while there was no significant difference in menopausal age in the obese and nonobese groups (Table 1).

The incidence of metabolic syndrome was much higher in the obese groups in both genders, and the risk of metabolic syndrome increased 5.86-fold in the obese group (*p* < 0.001; Table 1). The components of metabolic syndrome were higher in the obese group than the nonobese group in both genders. Serum total cholesterol and LDL concentrations were much higher in women than men, but the serum triglyceride concentrations were higher in men than women (Table 1). The serum HDL concentrations were higher in women than men. In the combined lipid profiles, women had as many lipid disturbances as men. Furthermore, obese women exhibited higher serum hs-CRP concentrations than nonobese women, but this was not observed in men (Table 1). Blood pressure, including systolic blood pressure (SBP) and diastolic blood pressure (DBP), was higher in the obese group than the nonobese group, and men showed higher blood pressure than women. Women had much higher eGFR than men, and eGFR was lower in the obese group than in the nonobese group. The serum alanine aminotransferase (ALT) and aspartate aminotransferase (AST) concentrations were higher in the obese groups than the nonobese groups in both genders. Obesity increased the odds ratio for higher AST and ALT concentrations by 2.0 and 2.7-fold, respectively (Table 1). 

### 3.2. Lifestyle Characteristics of the Participants According to Genders and Obesity

The daily energy intake based on the estimated energy intake was higher in the obese groups than the nonobese group in both genders, and it increased the obesity risk by 1.24-fold. The protein intake also elevated the obesity risk (*p* < 0.05; Table 2) based on the daily energy intake. In women, the carbohydrate intake was higher in the obesity group than the nonobese group, and the association of the carbohydrate intake with the obesity risk was marginally significant (*p* = 0.0508). Fat, including saturated, monounsaturated, polyunsaturated fatty acids, and cholesterol intake, were not associated with the obesity risk (Table 2). Their intake was slightly higher in the obese group than the nonobese group in men, but their intakes showed an opposite tendency in women. DII was similar in the obese and nonobese groups, but DII was higher in men than women (Table 2). Interestingly, the fried-food intake was higher in the obese group than the nonobese group in both genders, but the intake of sugar-containing foods was higher in the nonobese groups than the obese group in women, but not men (Table 2). Obese men had a higher intake of a Korean balanced diet and Western-style diet than the nonobese men, while there was no difference in the plant-based diet and rice-based diet intake in obese and nonobese men (Table 2). Obese women had a lower plant-based diet and a higher Korean balanced diet than nonobese women. There was no significant association of dietary patterns with obesity risk (Table 2).

Alcohol intake was much higher in the obese group than the nonobese group in both genders, particularly in men (Table 2). Alcohol intake was positively associated with the obesity risk. Fewer current smokers belonged to the obese group than the nonobese group in men but not women (Table 2). Regular exercise lowered the incidence of obesity in women but not men. Regular exercise was inversely associated with the risk of obesity (Table 2). 

### 3.3. Genetic Variants Related to the Obesity Risk and the Best Model with SNP-SNP Interaction

Ten genes with SNPs that affect the obesity risk were chosen to examine genetic variant−genetic variant interactions in the GMDR in the urban hospital-based cohort (Table 3). The selected genetic variants involved in the obesity risk were SEC16 Homolog B *(SEC16B*, endoplasmic reticulum export factor) rs543874, DnaJ heat shock protein family (Hsp40) member C27 (*DNAJC27)*_rs713586, CDK5 regulatory subunit-associated protein 1-like 1 (*CDKAL1)*_rs9356744, transcription factor AP-2 Beta *(TFAP2B)*_rs2206277, *BDNF*_rs6265, myosin light chain-2 (*MYL2)*_rs3782889, olfactomedin-4 (*OLFM4)*_rs9568856, *FTO*_rs1421085, *MC4R*_rs6567160, and *GIPR*_rs1444988703 (Table 3). These genes were strongly connected, and *FTO* and *MC4R* acted as hub genes that affect the risk of obesity [5]. All of the genes were involved in either energy intake or energy expenditure by modulating transcription factors. These SNPs satisfied the MAF (>0.01) and HWE (*p* > 0.05) criteria (Table 2). Some SNPs had an inverse association with obesity risk, while the other SNPs were positively associated with the obesity risk. The *p* values of most selected SNPs were higher than 5×10^−7^, but rs3782889_*MYL2* and rs9356744_*CDKAL1* did not meet the statistical criteria (Table 3). These SNPs had similar associations with the obesity risk, and the statistical significance was higher in the Ansan/Ansung cohort (Table 3), but they were not ultimately included in the best model.

Among the genetic variants selected, the models including 5, 7, 8, 9, and 10 SNPs met the criteria of TREB and CVC for the best model to explain the SNP−SNP interactions that contribute to obesity risk. The five models exhibited significant interactions among the genetic variants that influenced the obesity risk (Table 4). The 5-SNP model included *SEC16B*_rs543874, *DNAJC27*_rs713586, *BDNF_*rs6265, *MC4R*_rs6567160, and *GIPR_*rs1444988703. The genetic variants in model 5 plus *CDKAL1*_rs9356744 and *OLFM4*_rs9568856 (model 7) also exhibited significant interactions (Table 4). The models that added the remainder of the SNPs to model 7 also had significant interactions for obesity risk. After adjusting for covariates, the models included five, seven, eight, nine, and ten genetic variants that met the TRBA, TEBA, and CVC criteria, including gender, age, resident area, regular exercise, and smoking status. However, since all of these models have similar statistical significance; therefore model 5, which included the smallest number of variables, was selected.

**Figure 2:**
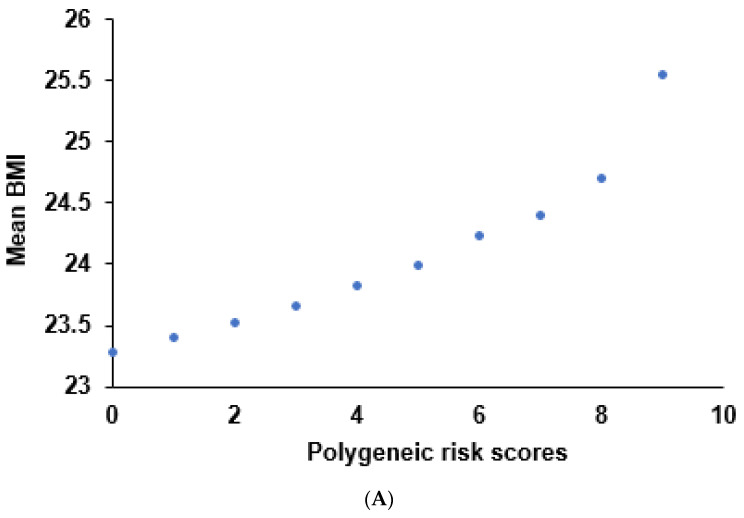

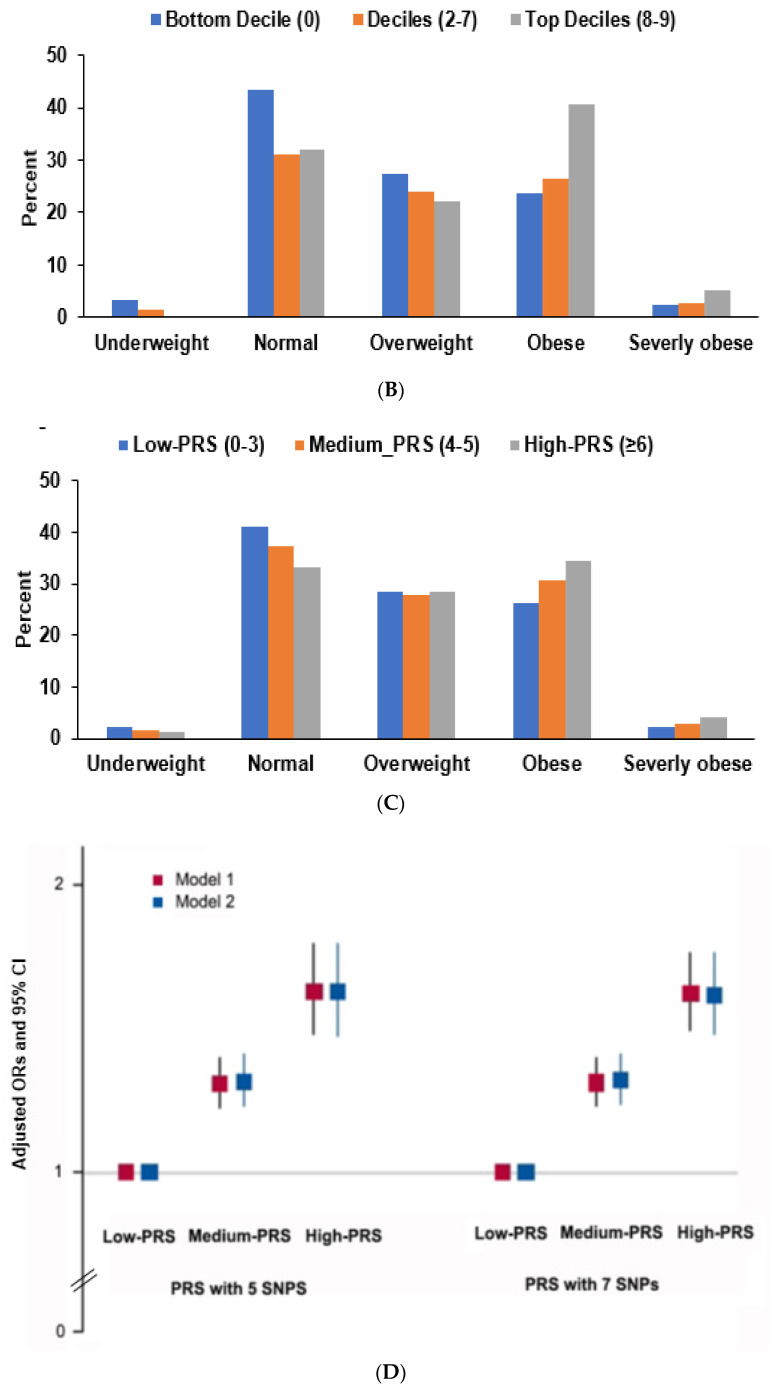
Relationship of polygenetic risk scores (PRS) to obesity risk: (**A**) BMI of participants according to 10 deciles of PRS; (**B**) the percentage of the participants according to obesity and bottom (0), middle (1–7), and top (8–9) deciles of PRS; (**C**) the percentage of the participants according to obesity and low (0–3), medium (4–5), and high (≥6) PRS in the 5-SNP model; (**D**) adjusted odds ratios (ORs) and 95% confidence intervals (CI) of the PRSs of 5- and 7-SNP models were generated by assessing gene−gene interactions associated with obesity risk. PRSs of the 5- and 7-SNPs were calculated by summing the number of risk alleles of SNPs. PRS of the 5-SNP model included 0–9 deciles, and it was divided into the bottom (0), middle (1–7), and top (8–9) in B. In C, PRS calculated using the 5- and 7-SNPs models were divided into three categories (0–3, 4–5, and ≥6) and (0–5, 6–7, and ≥8), respectively. Underweight refers to BMI < 18.5 kg/m^2^, normal as 18.5 to 23 for men 18.5 to 22 kg/m^2^ for women, overweight as 25.0 to 29.9 kg/m^2^, obesity as 30.0 to 39.9 kg/m^2^, and severe obesity as ≥40 kg/m^2^. Adjusted ORs were obtained by logistic regression using age, gender, education, income, occupation, residence area, and energy intake (percentage of estimated energy requirement) (model 1), plus variables in model 1, regular exercise, alcohol intake, and smoking status as covariates. The lowest category PRS were used as reference scores for logistic regression. Red and blue boxes indicate adjusted ORs for the 5- and 7-SNP models, respectively, and lines on these boxes indicated 95% CI.

Model 5 resulted in participants having PRS scores ranging from 0–9 risk alleles. The results show that BMI showed an increasing trend with PRS, and the participants with PRS scores of 8 and 9 had a sharp increment in BMI (Figure 2A). Although there was a positive association between PRS and BMI, some severely obese and underweight participants were distributed among the top deciles of PRS (8 and 9) to the bottom decile (0). However, obese (25–30 kg/m^2^) and severely obese (>30 kg/m^2^) persons had much higher representations in the middle deciles (2–7) than normal (18.5–23 kg/m^2^ for men and 18.5–22 kg/m^2^ for women), overweight (23–25 kg/m^2^ for men and 22–25 kg/m^2^ for women), and underweight (Figure 2B). In the other stratification, participants were assigned to low PRS (0–3), medium PRS (4–5), and high PRS (≥6) (Figure 2C). The PRS stratification showed a similar pattern of the top, middle, and bottom deciles, but the percentage of overweight participants in the three PRS groups was not significantly different.

The high PRS of the 5-SNP model was positively associated with the obesity risk: by 1.626 (1.474–1.801) and 1.629 (1.475–1.798) times after adjusting for covariates 1 and 2, respectively (Figure 2D). The high PRS with seven SNPs also showed a positive association with the obesity risk, but the ORs of the 7-SNP model were slightly smaller than those in the 5-SNP model (Figure 2D).

The PRS was significantly associated with the BMI: 1.43-fold and 1.55-fold in men and women, respectively, after adjusting for the covariates (Table 5). On the other hand, PRS was not related to waist circumference and SMI after adjusting for the covariates, including BMI. Interestingly, the fat mass was positively associated with fat by 1.46-fold only in men but not women after adjusting for covariates, including the BMI (Table 5). In addition, PRS was not related to menarche and menopausal age in women after adjusting for the covariates. 

### 3.4. Interaction of PRS and Nutrient Intake in Obesity Risk

**Figure 3:**
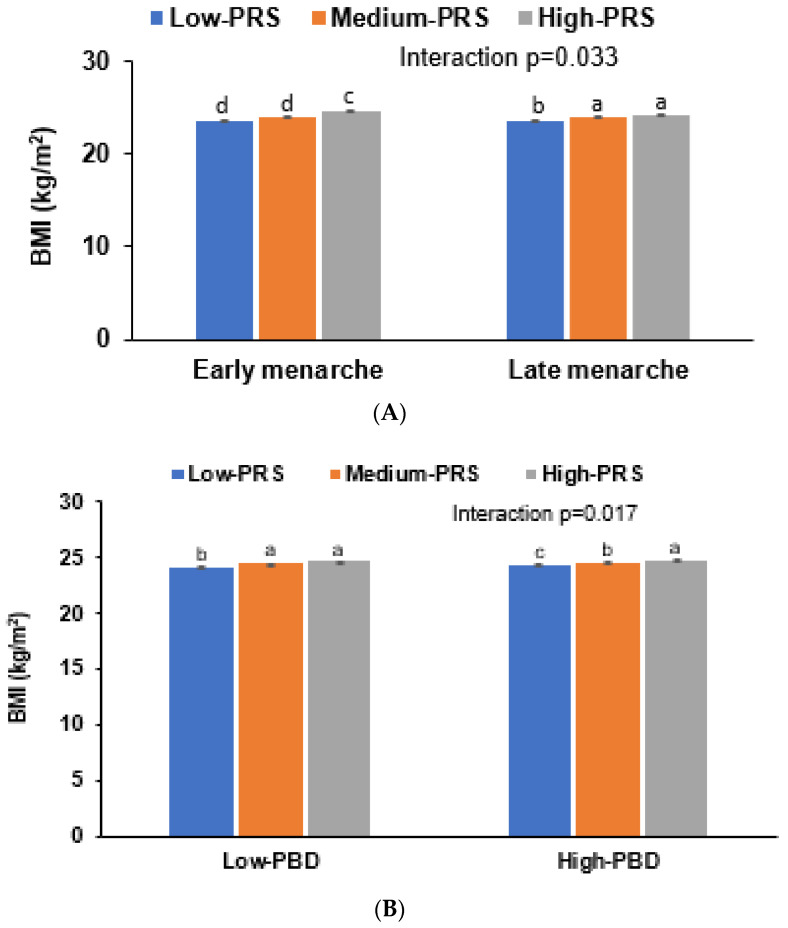

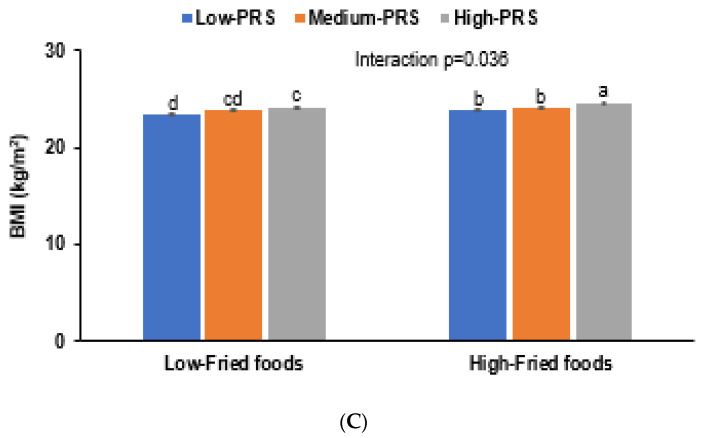
Body mass index (BMI) of participants with low, medium, or high polygenetic risk scores (PRS) as determined using the 5-SNP model. (**A**) Adjusted means and standard errors of participants of BMI categorized by menarche age (a cutoff value: 15 years). (**B**) Adjusted means and standard errors of participants of BMI categorized by a plant-based diet (PBD; a cutoff value: 70th percentiles). (**C**) Adjusted means and standard errors of participants of BMI categorized by fried-food intake (a cutoff value: once a week). Covariates included age, gender, education, income, energy intake (percentage of estimated energy requirement), occupation, residence area, regular exercise, alcohol intake, and smoking status. ^a–d^ Different letters on the bar indicated significant differences among the groups in Tukey’s test at *p* < 0.05.

Interestingly, initial menstruation age interacted with PRS for the obesity risk. In participants with an early menarche age, the BMI was higher than those with late menarche age. The impact of PRS was much higher in the participants with early menstruation than those with late menstruation (*p* = 0.027; Figure 3A). The adjusted ORs and 95% CI were 2.38 and 1.608–2.841 in the participants with early menstruation and 1.562 and 1.405–1.738 in those with late menstruation, respectively (Table 6).

A plant-based diet intake showed an interaction with the PRS to influence the obesity risk (Table 6). In a low plant-based diet intake, the genetic impact was lower for BMI, whereas the participants with a high PRS had a higher BMI than those with a low PRS with a high plant-based dietary intake (Figure 3B). Adults with a high PRS were protected against the obesity risk in a high plant-based diet (Table 6). The nutrient intake and other dietary patterns did not show any interactions with the PRS to affect the obesity risk.

Fried-food intake exhibited an interaction with the PRS (Table 6). The high fried-food intake participants had a higher BMI, but the PRS impact was offset in the participants with a high fried-food intake (Figure 3C). Thus, fried-food intake should be avoided to protect against obesity, even though a high PRS did not significantly exacerbate the obesogenic effects of fried-food intake (*p* = 0.035).

## 4. Discussion

GWAS studies have previously identified genetic variants associated with obesity risk, but few studies have been conducted to identify the genetic variant−genetic variants and genetic variant−lifestyle interactions. The present study showed the participants with high PRS of appetite-related genes, mainly MC4R pathway-related genes, including *SEC16B*_ rs543874, *BDNF*_ rs6265 *DNAJC27*_ rs713586, *MC4R*_rs17782313, *GIPR*_ rs1444988703, increased the obesity risk by 1.6-fold. The participants with early menarche age, low plant-based diet, and high fried-food intake had a higher PRS impact on the obesity risk than those with late menarche age, high plant-based diet, and low fried-food intake. Therefore, the participants with high PRS, especially with early menstruation, could be at obesity risk, and they should consume a plant-based diet with fewer fried foods to prevent obesity risk.

The arcuate nucleus of the hypothalamus, a central organ for regulating energy balance, has a range of receptors for appetite-regulating hormones and neurotransmitters. Leptin, insulin, ghrelin, cholecystokinin, and glucagon-like peptide-1 modulate the activation of orexigenic neurons and anorexigenic neurons of the hypothalamus [31]. When the hunger center is activated, food intake increases and energy expenditure decreases. The leptin−melanocortin signaling system is the critical appetite regulatory system: leptin activates proopiomelanocortin, an α-melanocyte-stimulating hormone that activates the melanocortin type 3 (MC3R) and MC4R. MC4R stimulates the anorexigenic effectors and suppresses the orexigenic effectors [32]. Therefore, the MC4R-related pathway is involved in obesity risk.

The present study shows that MC4R mutations are reported to develop obesity ranging from 0.5–5.8% [32]. A GWAS study in Danish, Icelandic, Dutch, European Americans, and African American adults has shown that SNPs in the *FTO*, *MC4R*, *BDNF*, and *SH2B1* genes are highly associated with obesity risk via modulating leptin secretion to induce leptin resistance in different ethnicities [33,34,35]. In Chinese people, *SEC16B* is involved in the serum leptin concentration connected with *FTO*, *MC4R*, and *BDNF* involved in the appetite regulation in the hypothalamus [34]. *SEC16B* is expressed in various tissues and is also involved in transporting appetite-regulatory peptides, including neuropeptide Y and proopiomelanocortin [36]. It acts in the adipose tissues to secrete obesity-related molecules that regulate fat mass [37]. Adults with the risk alleles of *MC4R*_rs17782313 have higher ghrelin concentrations, and more than 50% have severe binge eating issues [38]. The genes and genetic variants were associated with high BMI in middle-aged adults and the elderly. Furthermore, afamelanotide stimulates *BDNF* expression in the brain by activating *MC4R* to mediate neurogenesis and cognitive function in an Alzheimer’s disease animal model [35]. The present study showed that *MC4R*_rs17782313 and *BDNF*_rs6265 were included in the best genetic model for obesity. The two SNPs might interact with each other to influence the obesity risk through modulating appetite.

This study demonstrates that genetic factors can interact with environmental factors in obesity development, and obesity can be prevented with high compliance using genetic and environmental interaction results. The association of fat and carbohydrate intake with obesity remains controversial, although the daily energy intake was positively associated with obesity risk. Furthermore, food intake has shown controversial results for obesity risk. The present study reported that DII, a dietary inflammatory index, and sugar-containing food intake were not involved in the obesity risk, while fried-food intake was positively associated. In contrast, the serum hs-CRP concentrations have a positive association with obesity. Therefore, obesity may be associated with inflammation, but the DII itself may not fully represent the inflammation status in the present study.

Dietary patterns have been reported to influence obesity risk [39,40,41,42]. A plant-based diet was inversely associated with the obesity risk, whereas a Western-style diet had a positive association in the present study. In the present study, the participants with a high intake of plant-based foods had a higher energy intake with a high intake of fat and low intakes of carbohydrates. Thus, the intake of a plant-based diet might reduce the obesity risk despite the higher energy intake. The previous study has also shown that a plant-based diet is inversely related to liver fat deposition in children aged 6 to 15 [39]. These results suggest that the reduced obesity risk may be related to the gut microbiome because a high plant-based diet had a higher dietary fiber intake, but a Western diet had a lower fiber intake. Previous studies [40,41,42] reported that the participants with vegetarian-type diets have more of the *Prevotella* enterotype in the Western countries, whereas among Koreans, those with a rice-based diet have a higher level of *Prevotella*. Therefore, a plant-based diet may be intermediate between a rice-based diet and a balanced Korean diet. Thus, a plant-based diet improves obesity risk potentially by promoting the gut microbiota balance.

A few studies have shown the genetic variants−diet interaction for obesity risk [43]. Previous studies have not shown that *MC4R*, *BDNF*, *SEC16B,* and *FTO* genetic variants interacted with the food and nutrient intake, even though nutrient interactions with the genetic variants of other genes, such as *GRB14*, *LYPLAL1*, *LRRN6C*, and *MTIF3,* have been reported [43]. In the present study, a plant-based diet and fried-food intake interacted with a high PRS, but other lifestyle-related factors did not interact with the PRS. The fried-food intake increased BMI, but the PRS impact was attenuated in the high intake group of fried foods, even though BMI was higher in the high intake group of fried food in the adults regardless of PRS. A high intake of fried foods has been shown to be related to hypertension, cardiovascular disease, and cancer risk [44,45,46]. Therefore, people with high PRS may have a higher risk of obesity and other metabolic disorders, and they need to consume less fried foods to reduce obesity risk.

The merit of this study was to show that the PRS of the appetite-related genetic variants increased obesity risk, and a high PRS had a higher impact on obesity with an early menarche age, high fried-food intake, and low plant-based diet intake. This result can be used as a basis of personalized nutrition for obesity risk. This study had some limitations. First, this was a case-control study that did not show a causal relationship. Second, although the results were analyzed from a large cohort, this study was not conducted with a survey design. Lifestyles and nutrient intake were self-reported, and they might include some biases. Finally, the PRS model employed in this study probably underestimates the true genetic impact on obesity since a limited number of genes were included. This model was clinically used as a tool in personalized nutrition and only included well-described SNPs associated with known metabolic pathways. Future research should be conducted with a total PRS that evaluates the complete impact of genetics on obesity among Koreans.

In conclusion, a PRS model using *SEC16B*_rs543874, *DNAJC27*_rs713586, *BDNF*_rs6265, *MC4R*_ rs6567160, and *GIPR*_rs1444988703 had gene−gene interactions which elevated the obesity risk. In addition, the PRS interacted with the menarche age in adult women with high PRS: those who had an early menarche age showed an elevated obesity susceptibility in later life. PRS also interacted with a plant-based diet and fried-food intake. Therefore, people with high PRS, especially women with early menarche, need to consume a more plant-based diet with fewer fried foods to decrease their obesity risk. These results can be applied to personalized nutrition for protecting against obesity from childhood.

## Figures and Tables

**Table 1:** Demographic and biochemical characteristics and association with obesity in the participants according to genders and obesity status.

	Men (*n* = 19,444)		Women (*n* = 34,384)		Adjusted ORs and 95% CI
	Control (*n* = 11,690)	Obese (*n* = 7754)	Control (*n* = 24,593)	Obese (*n* = 9791)	
Age (yr) ^1^	56.9 ± 0.08 ^a^	56.6 ± 0.10 ^a^	51.4 ± 0.05 ^d^	52.6 ± 0.08 ^c^***^###+++^	1.106 (1.030–1.188)
Height (cm) ^2^	168.6 ± 0.06 ^a^	168.4 ± 0.07 ^a^	156.9 ± 0.04 ^b^	156.2 ± 0.06 ^c^***^###+++^	1.013 (0.934–1.099)
BMI (mg/kg^2^) ^3^	22.7 ± 0.02 ^b^	27.0 ± 0.02 ^a^	22.2 ± 0.01 ^b^	27.2 ± 0.02 ^a^***	
Waist circumference (cm) ^4^	81.8 ± 0.07 ^c^	91.1 ± 0.08 ^a^	75.2 ± 0.05 ^d^	85.7 ± 0.07 ^b^***^###+++^	18.29 (16.46–20.34)
SMI (%) ^5^	29.8 ± 0.02 ^b^	32.9 ± 0.03 ^a^	23.4 ± 0.02 ^d^	25.9 ± 0.02 ^c^***^###+++^	3.380 (3.111–3.673)
Fat mass (%) ^6^	21.3 ± 0.02 ^d^	26.2 ± 0.03 ^c^	29.4 ± 0.02 ^b^	35.3 ± 0.03 ^a^***^###+++^	20.71 (17.83–24.16)
Menarche age ^7^			15.2 ± 0.01	15.0 ± 0.02 ***	0.778 (0.725–0.834)
Menopausal age ^8^			49.3 ± 0.04	49.3 ± 0.06	1.410 (0.975–2.038)
Education ≤Middle school High school ≥College	954 (13.2) 1431 (19.8) 4843 (67.0)	592 (12.9) 941 (20.4) 3070 (66.7)	3255 (17.6) 4004 (21.6) 11,254(60.8)	2296 (28.0) *** 2084 (25.5) 3809 (46.5)	1 0.812 (0.756–0.872) 0.627 (0.587–0.671)
Income ≤USD 2000 USD 2000–4000 >USD 4000	965 (8.68) 4936 (44.4) 5222 (47.0)	544 (7.38) *** 2900 (39.3) 3929 (53.3)	2338 (10.1) 9973 (43.0) 10,896(47.0)	1378 (15.1) *** 4299 (47.1) 3460 (37.9)	1 0.985 (0.915–1.061) 0.915 (0.854–0.980)
MetS (%) ^9^	942 (8.1)	2514(32.4) ***	1478 (6.0)	2725 (27.8) ***	5.860 (5.307–6.471)
Serum glucose (mg/dL) ^10^	96.4 ± 0.23 ^b^	100.4 ± 0.27 ^a^	92.3 ± 0.15 ^c^	97.2 ± 0.23 ^b^***^###+^	1.668 (1.548–1.796)
HbA1c (%) ^11^	5.61 ± 0.01 ^d^	5.80 ± 0.01 ^b^	5.66 ± 0.01 ^c^	5.87 ± 0.01 ^a^***^###^	1.695 (1.517–1.893)
Serum total cholesterol ^12^	188.5 ± 0.41 ^d^	192.1 ± 0.48 ^c^	200.4 ± 0.27 ^b^	204.8 ± 0.41 ^a^***^###^	1.475 (1.352–1.610)
Serum HDL ^13^	51.0 ± 0.15 ^c^	46.3 ± 0.17 ^d^	57.7 ± 0.10 ^a^	53.0 ± 0.14 ^b^***^###^	1.913 (1.756–2.083)
Serum LDL ^14^	113.0 ± 0.38 ^c^	113.8 ± 0.45 ^c^	120.3 ± 0.25 ^b^	124.4 ± 0.37 ^a^***^###+++^	1.484 (1.338–1.647)
Serum Triglyceride ^15^	122.6 ± 0.97 ^b^	160.6 ± 1.14 ^a^	111.8 ± 0.64 ^d^	137.3 ± 0.96 ^c^***^###+++^	2.157 (2.006–2.320)
Serum hs-CRP ^16^	0.17 ± 0.01 ^ab^	0.19 ± 0.01 ^a^	0.12 ± 0.02 ^b^	0.23 ± 0.03 ^a^***^+^	1.266 (1.008–1.589)
SBP (mmHg) ^17^	123.7 ± 0.16 ^c^	128.9 ± 0.19 ^a^	119.0 ± 0.11 ^d^	125.3 ± 0.16 ^b^***^###+++^	1.782 (1.657–1.916)
DBP (mmHg) ^18^	77.2 ± 0.11 ^b^	80.5 ± 0.13 ^a^	73.1 ± 0.07 ^c^	76.9 ± 0.11 ^b^***^###++^	1.946 (1.750–2.164)
eGFR (ml/min) ^19^	84.7 ± 0.18 ^c^	83.3 ± 0.24 ^d^	86.9 ± 0.13 ^a^	88.0 ± 0.21 ^b^***^+++^	1.140 (1.039–1.251)
Serum AST (U/L) ^20^	24.3 ± 0.25 ^b^	26.6 ± 0.31 ^a^	22.3 ± 0.17 ^c^	24.4 ± 0.27 ^b^***^###^	2.013 (1.837–2.205)
Serum ALT(U/L) ^21^	23.8 ± 0.24 ^b^	30.8 ± 0.29 ^a^	18.6 ± 0.16 ^c^	23.9 ± 0.26 ^b^***^###++^	2.724 (2.566–2.892)
Serum hs-CRP (mg/L) ^22^	0.17 ± 0.01 ^ab^	0.19 ± 0.01 ^a^	0.12 ± 0.02 ^b^	0.23 ± 0.03 ^a^**^+^	1.645 (1.201–2.254)

The values represent adjusted means ± standard deviations or the number of the subjects (percentage of each group). Covariates included age, gender, education, income, energy intake (percentage of estimated energy requirement), occupation, residence area, regular exercise, alcohol intake, and smoking status. The cutoff points of the reference for logistic regression were as follows: ^1^ < 55 years old for age, ^2^ <25 kg/m^2^ for BMI; ^3^ <172.5 cm for men and <160 cm for women; ^4^ < 90 cm for men and 85 cm for women waist circumferences; ^5^ <29.0% for men and 22.8% for women in skeletal muscle index (SMI defined as appendicular skeletal muscle mass/weight); ^6^< 25% for men and 30% for women for fat mass; ^7^ <14 years old; ^8^ <50 years old for menopause age; ^9^ Metabolic syndrome (MetS) criteria; ^10^ <126 mL/dL fasting serum glucose plus diabetic drug intake; ^11^ <6.5% HbA1c plus diabetic drug intake; ^12^ <230 mg/dL plasma total cholesterol concentrations; ^13^ >40 mg/dL for men and 50 mg/dL for women plasma HDL cholesterol; ^14^ <160 mg/dL plasma total cholesterol concentrations; ^15^ <150 mg/dL plasma triglyceride concentrations; ^16^ <0.5 mg/dL serum high-sensitive C-reactive protein (hs-CRP) concentrations; ^17^ <140 mmHg SBP, ^18^ < 90 mmHg DBP plus hypertension medication; ^19^ estimated glomerular filtration rate (eGFR) <70; ^20^ aspartate aminotransferase <40 U/L; ^21^ alanine aminotransferase <35 U/L; ^22^ high sensitive C-reactive protein <0.5 mg/d. ** Significant differences by obesity (BMI ≥ 25) at *p* < 0.01, *** *p* < 0.001. ### Significant differences by gender at *p* < 0.001. + Significant interaction between gender and obesity at *p* < 0.05, ++ at *p* < 0.01, +++ *p* < 0.001. ^a–d^ values with different superscript letters in the same row were significantly different by Tukey’s test at *p*<0.05.

**Table 2:** Lifestyles including nutrient intake and association with obesity in the participants according to genders and obesity status.

	Men (*n* = 19,444)		Women (*n* = 38,384)		Adjusted ORs and 95% CI ^1^
	Control (*n* = 11,690)	Obese (*n* = 7754)	Control (*n* = 24,593)	Obese (*n* = 9791)	
Energy (<EER %) ^2^	90.2 ± 0.32 ^3^	92.4 ± 0.39	92.8 ± 1.07	103.5 ± 1.62 ***^###+++^	1.244 (1.153–1.342)
CHO (<70 En %)	71.0 ± 0.07 ^a^	70.7 ± 0.09 ^a^	69.6 ± 0.26 ^b^	70.1 ± 0.39 ^ab###^	0.946 (0.895–1.000)
Protein(<14 En%)	13.6 ± 0.03 ^b^	13.5 ± 0.03 ^b^	14.2 ± 0.10 ^a^	14.1 ± 0.15 ^a###^	1.047 (0.998–1.090)
Total fat (<15 En%)	14.5 ± 0.06 ^b^	14.6 ± 0.07 ^b^	15.4 ± 0.20 ^a^	14.9 ± 0.30 ^ab##^	1.020 (0.977–1.064)
Saturated fat (<4.7 En%)	0.44 ± 0.002 ^b^	0.46 ± 0.003 ^a^	0.45 ± 0.002 ^a^	0.44 ± 0.003 ^b+++^	1.032 (0.986–1.080)
Monounsaturated fat (<6.0 En%)	0.56 ± 0.003 ^b^	0.58 ± 0.004 ^a^	0.55 ± 0.002 ^c^	0.54 ± 0.003 ^d###+++^	1.001 (0.955–1.049)
Polyunsaturated fat (2.5 En%)	0.32 ± 0.003 ^ab^	0.33 ± 0.003 ^a^	0.31 ± 0.002 ^b^	0.31 ± 0.003 ^b###++^	1.038 (0.992–1.087)
Cholesterol (<200 mg/d)	179 ± 1.13 ^a^	181 ± 1.33 ^a^	165 ± 0.74 ^b^	162 ± 1.12 ^c###++^	0.985 (0.930–1.043)
Fiber (6 g/d)	5.98 ± 0.02 ^a^	5.94 ± 0.03 ^a^	5.51 ± 0.02 ^b^	5.49 ± 0.02 ^b###^	0.985 (0.892–1.086)
DII (<2374 scores)	2096 ± 15.9 ^a^	2088 ± 18.8 ^a^	1917 ± 10.5 ^b^	1939 ± 15.9 ^b###^	0.980 (0.933–1.030)
Fried foods (<0.6/week)	0.53 ± 0.01 ^b^	0.60 ± 0.01 ^a^	0.42 ± 0.01 ^c^	0.50 ± 0.01 ^b^***^###^	1.217 (1.117–1.326)
Sugar-containing foods	3.05 ± 0.09 ^a^	2.98 ± 0.10 ^a^	2.79 ± 0.06 ^a^	2.45 ± 0.09 ^b^**^##^	0.984 (0.905–1.070)
Balanced Korean diet (<70th percentile)	10,984 (66.9)	2114 (69.9) **	19,746 (64.6)	2843(67.2) **	1.137 (1.089–1.186)
Plant-based diet (<70th percentile)	8721 (53.1) ^4^	1552 (51.3)	21,961 (72.8)	2857 (67.5) ***	0.868 (0.832–0.907)
Western-style diet (<70th percentile)	12,949 (78.9)	2487 (82.2) ***	17,898 (59.4)	2552 (60.3)	1.142 (1.092–1.195)
Rice-based diet (<70th percentile)	10,949 (66.7)	1974 (65.2)	19,580 (64.9)	2828 (66.8) *	1.001 (0.960–1.045)
Alcohol drinking (<100 g/week)	199 ± 3.37 ^b^	241 ± 3.96 ^a^	57.8 ± 2.22 ^d^	64.1 ± 3.36 ^c^***^###+++^	1.139 (1.060–1.225)
Smoking status (current smokers)	3423 (29.4)	2106 (27.2) ***	469 (1.91)	212 (2.17)	0.820 (0.761–0.884)
Regular Exercise ^5^	6897 (59.0)	4575 (59.0)	12,961 (52.7)	4523 (46.2) ***	0.444 (0.203–0.974)

^1^ Odds ratio (ORs) and 95% confidence intervals (CI) in logistic regression after adjusting for covariates included age, gender, education, income, energy intake (percentage of estimated energy requirement), occupation, residence area, regular exercise, alcohol intake, and smoking status. ^2^ The cutoff points for logistic regression. ^3^ The values represent adjusted means ± standard deviations. ^4^ The number of the subjects (percentage of each group). ^5^ The cutoff points of regular exercise for logistic regression were as follows: 30 min of moderate exercise 3 times per week when moderate exercise was defined as the exercise corresponding to 3 ≤ metabolic equivalents of task (METs) ≤ 6. * Significant differences by gender at *p* < 0.05, ** at *p* < 0.01, *** *p* < 0.001. ^##^ Significant differences by obesity (BMI ≥ 25) at *p* < 0.01, ^###^ *p* < 0.001.^++^ Significant interaction between gender and obesity at *p* < 0.01, ^+++^ *p* < 0.001. ^a–d^ values with different superscript letters in the same row were significantly different by Tukey’s test at *p* < 0.05.

**Table 3:** Characteristics of genetic variants mainly related to appetite regulation for obesity risk.

Chr ^1^	SNP ^2^	Position	Mi ^3^	Ma ^4^	OR and 95% CI for City ^5^	*p*-Value Adjusted (City) ^6^	*p*-Value Adjusted (Urban) ^7^	MAF ^8^	*p*-Value for HWE ^9^	Gene	Functional Consequence
1	rs543874	177889480	G	A	1.13 (1.10–1.17)	6.65 × 10^−16^	1.52 × 10^−3^	0.249	0.581	*SEC16B*	exon
2	rs713586	25158008	C	T	1.08 (1.05–1.11)	2.16 × 10^−8^	2.25 × 10^−3^	0.485	0.225	*DNAJC27*	exon
6	rs9356744	20685486	C	T	0.95 (0.93–0.98)	4.06 × 10^−5^	8.80 × 10^−5^	0.466	0.102	*CDKAL1*	intron
6	rs2206277	50798526	T	C	1.07 (1.04–1.11)	4.35 × 10^−7^	5.06 × 10^−2^	0.310	0.969	*TFAP2B*	intron
11	rs6265	27679916	T	C	0.92 (0.89–0.94)	2.04 × 10^−10^	2.84 × 10^−1^	0.459	0.148	*BDNF*	missense
12	rs3782889	111350655	G	A	0.94 (0.91–0.97)	4.84 × 10^−4^	8.11 × 10^−3^	0.171	0.449	*MYL2*	intron
13	rs9568856	54064981	A	G	1.08 (1.05–1.11)	1.33 × 10^−7^	3.17 × 10^−1^	0.285	0.620	*OLFM4*	intron
16	rs1421085	53800954	C	T	1.18 (1.13–1.22)	1.82 × 10^−16^	1.54 × 10^−6^	0.125	0.460	*FTO*	intron
18	rs17782313	57829135	C	T	1.11 (1.08–1.15)	3.16 × 10^−12^	2.14 × 10^−4^	0.239	0.585	*MC4R*	exon
19	rs1444988703	46175046	A	T	1.10 (1.07–1.13)	2.86 × 10^−12^	6.22 × 10^−5^	0.407	0.441	*GIPR*	intron

^1^ Chromosome; ^2^ single nucleotide polymorphism; ^3^ minor allele; ^4^ major allele, ^5^ odds ratio (OR) and 95% confidence intervals (CI) for city cohort; ^6^ *p*-value for OR after adjusting for age, gender, residence area, survey year, body mass index, daily energy intake, education, income and regular exercise in the city cohort; ^7^ *p*-value for OR for Ansan/Ansung cohort after adjusting the covariates; ^8^ minor allele frequency; ^9^ Hardy−Weinberg equilibrium.

**Table 4:** Generalized multifactor dimensionality reduction (GMDR) results of multilocus interaction with genes mainly related to appetite regulation for obesity risk.

GMDR	Adjusted for Gender, Age, and Residence Area				Adjusted for Gender, Age, Residence Area, Regular Exercise, and Smoking Status			
Model	TRBA	TEBA	*p*-Value	CVC	TRBA	TEBA	*p*-Value	CVC
*SEC16B*_ rs543874	0.5171	0.5144	10 (0.0010)	8/10	0.5174	0.5156	10 (0.0010)	9/10
Model 1 plus *BDNF*_ rs6265	0.5237	0.5178	10 (0.0010)	6/10	0.5239	0.5179	10 (0.0010)	6/10
model 2 plus *GIPR*_ rs1444988703	0.5275	0.5180	10 (0.0010)	4/10	0.5276	0.5183	10 (0.0010)	4/10
Model 3 plus *FTO*_ rs1421085	0.5322	0.5228	10 (0.0010)	6/10	0.5326	0.5222	10 (0.0010)	4/10
**Model 3 plus *DNAJC27*_** rs713586, ***MC4R*_** rs17782313	**0.5413**	**0.5260**	**10 (0.0010)**	**10/10**	**0.5416**	**0.5254**	**10 (0.0010)**	**10/10**
Model 5 plus *OLFM4*_ rs9568856	0.5536	0.5232	10 (0.0010)	9/10	0.5539	0.5210	10 (0.0010)	6/10
**Model 6 plus *CDKAL1*_** rs9356744	**0.5774**	**0.5224**	**10 (0.0010)**	**10/10**	**0.5778**	**0.5197**	**10 (0.0010)**	**10/10**
**Model 7 plus *TFAP2B_*** rs2206277	**0.6129**	**0.5165**	**10 (0.0010)**	**10/10**	**0.6139**	**0.5145**	**10 (0.0010)**	**10/10**
**Model 8 plus *MYL2*_** rs3782889	**0.6550**	**0.5163**	**10 (0.0010)**	**10/10**	**0.6568**	**0.5163**	**10 (0.0010)**	**10/10**
**Model 4 plus *DNAJC27*, *CDKAL1, TFAP2B*, *MYL2*, *OLFM4*, *MC4R***	**0.6978**	**0.5129**	**10 (0.0010)**	**10/10**	**0.6996**	**0.5122**	**10 (0.0010)**	**10/10**

TRBA, trained balanced accuracy; TEBA, test balance accuracy; CVC, cross-validation consistency; sign test, *p*-value for the significance of GMDR model by sign test with and without adjusting for covariates designated in the table; BMI, body mass index. Boldface values indicated qualifying for the best model.

**Table 5:** Association of polygenetic risk scores (PRS) with metabolic parameters in the participants according to genders and obesity status.

	Men			Women	
	Low PRS ^1^	Medium PRS (*n* = 13,024)	High PRS (*n* = 1931)	Medium PRS (*n* = 23,094)	High PRS (*n* = 3344)
Age (<55 year)	1	0.965 (0.893–1.043)	1.019 (0.901–1.151)	0.986 (0.926–1.049)	0.975 (0.883–1.076)
Waist circumference (M: 95; F 85 cm)	1	0.873 (0.772–1.000)	0.907 (0.764–1.077)	0.971 (0.881–1.069)	0.886 (0.764–1.027)
BMI (<25 mg/kg^2^)	1	1.252 (1.161–1.350)	1.430 (1.272–1.608)	1.278 (1.199–1.362)	1.554 (1.412–1.711)
BMI (<27 mg/kg^2^)	1	1.232 (1.109–1.369)	1.479 (1.267–1.727)	1.375 (1.255–1.506)	1.742 (1.531–1.983)
Skeletal muscle index ^2^ (%)	1	0.976 (0.897–1.063)	0.940 (0.822–1.075)	0.998 (0.937–1.063)	0.928 (0.839–1.026)
Fat mass (%)	1	1.233 (1.136–1.338)	1.463 (1.291–1.657)	0.893 (0.785–1.015)	0.824 (0.656–1.001)
Metabolic syndrome (No)	1	1.062 (0.956–1.181)	1.106 (0.939–1.304)	1.006 (0.915–1.106)	0.892 (0.774–1.028)
Serum glucose (<126 mg/dL)	1	1.012 (0.936–1.094)	0.972 (0.859–1.099)	0.947 (0.881–1.017)	1.063 (0.952–1.187)
HbA1c (<6.5%)	1	1.021 (0.905–1.151)	1.058 (0.879–1.274)	1.009 (0.897–1.136)	1.182 (1.002–1.401)
Serum total cholesterol (<230 mg/dL)	1	0.884 (0.805–0.970)	0.826 (0.710–0.961)	1.012 (0.948–1.081)	1.055 (0.953–1.169)
Serum HDL (M: 40 F: 50 mg/dL)	1	1.006 (0.917–1.103)	0.992 (0.858–1.147)	1.031 (0.938–1.132)	1.007 (0.949–1.070)
Serum LDL (<140 mg/dL)	1	0.925 (0.827–1.034)	0.915 (0.767–1.092)	0.986 (0.915–1.063)	1.018 (0.906–1.144)
Serum triglyceride (<150 mg/dL)	1	0.931 (0.862–1.006)	0.869 (0.769–0.982)	0.942 (0.880–1.007)	1.030 (0.928–1.142)
SBP (<130 mmHg)	1	1.029 (0.954–1.110)	0.975 (0.865–1.100)	0.997 (0.935–1.063)	1.011 (0.916–1.117)
DBP (<90 mmHg)	1	0.977 (0.875–1.090)	1.026 (0.865–1.217)	0.999 (0.894–1.117)	1.071 (0.905–1.268
eGFR (<70 mL/min)	1	1.070 (0.879–1.304)	1.206 (0.898–1.619)	0.971 (0.817–1.154)	1.127 (0.869–1.462)
Serum hs-CRP (<0.5 mg/L)	1	1.606 1.106 2.332	1.719 (1.020–2.896)	0.491 (0.110–2.193)	0.613 (0.059–6.409)
Menarche age (<14 yr)	1			0.997 (0.926 1.074)	1.014 (0.903–1.140)
Menopausal age (<50 yr)	1			1.060 (0.995 1.129)	1.118 (0.998–1.242)

Values represent adjusted odds ratios and 95% confidence intervals. Covariates included age, gender, body mass index (BMI), education, income, energy intake (percentage of estimated energy requirement), occupation, residence area, regular exercise, alcohol intake, and smoking status. ^1^ PRS with 5 SNPs of the best GMDR model was divided into three categories according to the number of the risk alleles: when the number of risk alleles in the PRS was ≤ 3, 4–5, and ≥ 6 into low PRS, middle PRS, and high PRS, respectively. <29.0% for men and 22.8% for women in skeletal muscle index (SMI; defined as appendicular skeletal muscle mass/weight); reference was the low PRS (men: *n* = 4485; women: *n* = 7939). ^2^ Skeletal muscle mass divided by BMI; SBP, systolic blood pressure; DBP, diastolic blood pressure; eGFR, estimated glomerular filtration rate; hs-CRP, high-sensitive C-reactive protein.

**Table 6:** Adjusted odds ratios for obesity risk by polygenetic risk scores (PRS) of the best model for gene−gene interaction after covariate adjustments according to lifestyles patterns.

	Low PRS (*n* = 7939)	Medium PRS (*n =* 23,094)	High PRS (*n* = 3344)	PRS−Lifestyle Interaction *p*-Value ^3^
Early menarche (<14 yr) ^2^	1	1.152 (0.990–1.341) ^1^	1.785 (1.427–2.233)	0.0174
Late menarche	1	1.283 (1.219–1.351)	1.479 (1.367–1.600)	
	Low PRS (12,424)	Medium PRS (*n* = 36,118)	High PRS (*n* = 5275)	PRS−lifestyle interaction *p*-value
Low plant-based diet (<70th percentile)	1	1.241 (1.138–1.353)	1.462 (1.279–1.670)	0.0273
High plant-based diet	1	1.268 (1.118–1.437)	1.392 (1.141–1.699)	
Low intake of fried food (<1 times/w)	1	1.288 (1.220–1.359)	1.472 (1.355–1.600)	0.0364
High intake of fried food	1	1.196 (1.072–1.335)	1.616 (1.374–1.902)	

PRS with 5 SNPs was divided into three categories according to the number of the risk alleles: when the number of risk alleles in the PRS was ≤3, 4–5, and ≥6 into low-PRS, medium-PRS, and high-PRS, respectively. The reference was the low PRS. ^1^ Values represent adjusted odds ratios (95% confidence intervals) after adjusting for covariates including age, gender, education, income, energy intake (percentage of estimated energy requirement), occupation, residence area, regular exercise, alcohol intake, and smoking status. ^2^ The cutoff points to divide the two groups. ^3^ Multivariate ANCOVA models include the corresponding main effects, interaction terms of main effects, and potential confounders such as age, gender, energy intake, residence area, metabolic syndrome, occupation, education, income, BMI, WBC, smoking status, coffee, alcohol, regular exercise, and any medication for inflammatory diseases.

## Data Availability

The raw data involved in this study will be available by the authors to any qualified researcher.

## Abbreviations

MetS: metabolic syndrome; KoGES, Korean Genome and Epidemiology Study; SQFFQ, semi-quantitative food frequency questionnaire; ORs, odds ratios; CI, 95% confidence intervals; BMI, body mass index; *FTO,* fat mass and obesity-associated protein; *MC4R*, melanocortin 4 receptor; *BDNF*, and brain-derived neurotrophic factor; GWAS, genome-wide association study; *GIPR,* gastric inhibitory polypeptide receptor; *LHCGR,* luteinizing hormone/choriogonadotropin receptor; *CALCR,* calcitonin receptor; *ADCY,* adenylate cyclase; *TMEM18,* transmembrane protein 18; AMPK, AMP kinase; PRS, polygenetic variants scores; SBP, systolic blood pressure; DBP, diastolic blood pressure; eGFR, estimated glomerular filtration rate; DII, dietary inflammatory index; GMDR, generalized multifactor dimensionality reduction; TRBA, trained balanced accuracy; TEBA, testing balanced accuracy; CVC, cross-validation consistency; ANOVA, analysis of variance; *DNAJC27,* DnaJ heat shock protein family member C27; *CDKAL1,* CDK5 regulatory subunit-associated protein 1-like 1; *TFAP2B*, transcription factor AP-2 beta; *MYL2*, myosin light chain-2; *OLFM4*, olfactomedin-4; ALT, alanine aminotransferase; AST, aspartate aminotransferase.
